# Applications of Solid-Phase Microextraction and Related Techniques

**DOI:** 10.3390/molecules30040827

**Published:** 2025-02-11

**Authors:** Hiroyuki Kataoka

**Affiliations:** Laboratory of Applied Analytical Chemistry, School of Pharmacy, Shujitsu University, Nishigawara, Okayama 703-8516, Japan; hkataoka@shujitsu.ac.jp; Tel.: +81-86-271-8342

In recent years, various high-performance analytical instruments have been developed with improved sensitivity and performance. However, most still struggle to directly process complex matrices, such as biological materials, environmental samples, and food, making sample pretreatment essential. In the analytical process from sampling to data analysis, sample pretreatment is not only vital for extracting, separating, and concentrating target analytes from complex matrices, but also enhances detection, improves sensitivity and accuracy, and reduces instrument maintenance and operating costs, all of which significantly impact the reliability and practicality of analytical methods. Thus, developing environmentally friendly and efficient green sample pretreatment techniques, and integrating them into various analytical instruments, have become major challenges.

Liquid–liquid extraction, a classical sample pretreatment technique, requires large amounts of sample and harmful organic solvents, making it time-consuming, labor-intensive, and expensive. To address these drawbacks, solid-phase extraction (SPE) was developed, and has been widely adopted due to its relatively simple and efficient operation, lower cost, reduced organic solvent consumption, and high concentration capacity.

As part of green analysis strategies, various microextraction techniques have been developed to minimize sample sizes and solvent usage while improving efficiency and automation. These include micro solid-phase extraction (μSPE), dispersive-μ-SPE, magnetic solid-phase extraction (MSPE), solid-phase microextraction (SPME), stir bar sorptive extraction (SBSE), microextraction with packed sorbent (MEPS), in-needle microextraction (INME), pipette tip solid-phase extraction (PT-SPE), and disposable pipette extraction (DPX). Among these, SPME is a simple and convenient sample pretreatment technique that integrates sampling, extraction, preconcentration, and desorption into a single step, enabling automation, miniaturization, high-throughput processing, and direct online coupling with analytical instruments. Its advantages include improved extraction efficiency, reduced sample handling, and lower costs for solvents and disposal. Since its introduction by Arthur and Pawliszyn [1] in the early 1990s, more robust fiber assemblies and coatings with higher extraction efficiency, selectivity, and stability have been commercialized, making SPME widely applicable in fields such as environmental monitoring, food analysis, forensic science, pharmaceuticals, and bioanalysis [2,3].

Additionally, new extraction geometries utilizing capillary tubes, magnetic stirrer bars, or thin films instead of fibers, have been developed, along with advanced polymer coatings, offering superior absorption/adsorption capacity and selectivity [3,4]. In these microextraction techniques, the enrichment capacity, sample processing ability, and selective extraction of target compounds are highly dependent on the coating material. To enhance extraction efficiency and selectivity, new adsorbents including monoliths, ionic liquids (ILs)/polymer ILs, restricted access materials, molecularly imprinted polymers (MIPs), graphene/graphene oxide (GO), carbon nanotubes (CNTs), inorganic nanoparticles, metal–organic frameworks (MOFs), crystalline covalent organic frameworks (COFs), and amorphous covalent organic polymers (COPs) have been continuously developed.

This Special Issue, titled “Applications of Solid-Phase Microextraction and Related Techniques”, features three peer-reviewed review articles and nine original research papers from groups worldwide. The topics include: studies on supramolecular materials such as MOFs and COFs [Contribution 1], hybrid graphene-based materials (GBMs) [Contribution 2], MIPs [Contribution 3] and other coating materials for microextraction; research utilizing gas chromatography–mass spectrometry (GC-MS) [Contributions 4–8], GC-electron capture detection (GC-ECD) [Contribution 9], high-performance liquid chromatography (HPLC) [Contribution 10], and LC-MS/MS [Contribution 11,12] as coupled analytical techniques. These also include applications such as GC-MS analysis of volatile organic compounds (VOCs) using multi-walled carbon nanotubes-ionic liquid/polyaniline adsorbents (MWCNT-IL/PANI) [Contribution 8] and MonoTrap^TM^ [Contribution 5] as adsorbents, headspace (HS)-SPME/GC-MS for VOC analysis using commercial fibers [Contribution 6,7], formaldehyde derivatization in water with SPME Arrow for GC-MS analysis [Contribution 8], MSPE GC-ECD analysis of pesticides in environmental water using magnetic persimmon leaf composites [Contribution 9], membrane solid-phase extraction (ME) HPLC analysis of sulfonamides in urine using a COP mixed matrix membrane [Contribution 10], biological monitoring of occupational exposures to anticancer drugs via μSPE coupled with ultra-performance liquid chromatography–tandem mass spectrometry (UHPLC-MS/MS) [Contribution 11], and simultaneous analysis of tobacco smoke exposure and stress biomarkers in saliva using in-tube (IT) SPME with a Supel-Q PLOT capillary for LC-MS/MS analysis [Contribution 12]. An overview of these studies is provided below.

SPME is a widely used sample preparation technique in environmental monitoring due to its simplicity, environmental friendliness, and extraction capacity. It is employed for the extraction, clean-up, and preliminary concentration of environmental pollutants. Supramolecular materials, with their high surface-to-volume ratio, controlled porosity, and tunable surface properties, have emerged as promising SPME coatings. These materials offer unique selectivity, three-dimensional structures, and flexible design, facilitating interactions between analytes and coatings through multiple oriented functional groups [Contribution 1]. By modifying linkers, nodes, and monomer units, 3D frameworks with tailored structures and surface chemistry can be designed, making them superior to other nanostructured materials such as alloys, silica, and carbon-based materials. Functionalization with phenyl, amino, and ionic groups has proven particularly effective in establishing multiple directional interactions, including hydrogen bonding, π–π interactions, electrostatic interactions, and van der Waals forces between analytes and coatings. As a result, extraction is driven by steric fit and complementarity between the sorbent and the target molecule, leading to enhanced selectivity and enrichment capabilities. Riboni et al. [Contribution 1] review the state of the art in SPME coatings based on MOFs, COFs, and supramolecular macrocycles such as cyclodextrins, calixarenes, and cavitands for environmental monitoring, while also discussing future challenges.

Graphene, a single-layer carbon allotrope, is widely used in energy generation, electronics, sensors, and other areas of materials science due to its flexibility, lightness, thermal and electrical conductivity, and mechanical resistance to high pressure [Contribution 2]. Its large surface area, honeycomb-patterned binding sites, delocalized π electrons, and monolayer structure make it particularly effective as an adsorbent in sample preparation techniques [Contribution 2]. GO can be chemically functionalized with various components, including ILs, silica derivatives, magnetic materials, MIPs, resins, deep eutectic solvents, and carbon-based biosorbents. These hybrid GBMs offer excellent versatility and modification potential, with their high surface area and functional groups making them ideal adsorbents for miniaturized extraction strategies. Their compatibility with environmentally friendly synthesis approaches, such as bio-based graphene materials, enables reduced adsorbent usage and the development of biodegradable materials, supporting green analytical strategies. Cardoso et al. [Contribution 2] review recent advances in hybrid GBMs for miniaturized solid-phase sample preparation techniques, discussing their typical characteristics, application trends, particularly in offline techniques such as SBSE, MEPS, PT-SPE, DPX, d-µ-SPE, and MSPE.

MIPs are stable, custom-made polymers with molecular recognition functions formed during synthesis, making them excellent “smart adsorbents” for selective sample preparation. The fabrication of MIPs involves three main steps: prepositioning recognition functions, polymerization, and template removal. First, a template-monomer complex is formed, followed by polymerization, which creates a polymer network with the template molecule embedded. In the final step, the template is removed, leaving behind cavities complementary to the target molecule [Contribution 3]. Meanwhile, IT-SPME [4] has gained attention as a “green extraction technique” due to its minimal use of organic solvents, reduced liquid waste, high throughput, compact size, online instruments compatibility, automation capabilities, and ability to operation continuously overnight. Kataoka et al. [Contribution 3] review recent advancements in MIP-based IT-SPME methods, which integrate the selectivity of MIPs with the efficiency of IT-SPME, and discuss their applications.

VOCs are present in various environments, and some have been identified as harmful. Ahn and Bae [Contribution 4] developed an HS-INME GC-MS method using a novel extraction device consisting of a needle inserted with an adsorbent-coated wire, enabling solvent-free sample extraction. A newly synthesized adsorbent, MWCNT–IL/PANI, produced by mixing aniline and MWCNTs in the presence of IL, followed by electrochemical polymerization, exhibits high thermal stability and can be reused up to 150 times without performance loss. This material offers an environmentally friendly, solvent-free extraction approach. Additionally, Dugheri et al. [Contribution 5] extracted VOCs using a vacuum-assisted HS-MonoTrapTM sampling system, which integrates a commercially available small monolith hybrid adsorption device, MonoTrapTM, into a vacuum-assisted extraction setup. The extracted VOCs were analyzed by GC-MS/olfactometry and applied to characterize VOC emission profiles from hot mix asphalt. Among the 35 hot mix asphalt samples analyzed, the major odor-active VOCs were primarily aldehydes, alcohols, and ketones.

Some VOCs produced by living organisms play important physiological roles. Song et al. [Contribution 6] analyzed the chemical composition and VOC content of female and male buds of *Trichosanthes anguina* L. (Cucurbitaceae), also known as *Trichosanthes cucunerina* L., using HS-SPME-GC-MS with a commercially available carboxen/polydimethylsiloxane/divinylbenzene (CAR/PDMS/DVB) fiber. Multivariate statistical analysis identified differences in VOC composition between female and male buds, revealing chemical distinctions in monoecious plants. These findings provide useful insights into plant sexual differentiation. Similarly, Rajendran et al. [Contribution 7] examined VOC production during the fermentation of the lactic acid bacteria *Levilactobacillus brevis* WLP672 (LB672) using HS-SPME-GC-MS with a CAR/PDMS/DVB fiber. They found that adding different amino acids, alone or in combination, to a specific medium influenced VOC production. These results contribute to the development of plant-based fermentation strategies for enhancing flavor in meat and dairy products.

Formaldehyde (FA) is widely used in manufacturing industries, including food processing, textiles, wood production, and cosmetics. However, human exposure to FA can cause both acute and chronic toxicity, necessitating the need for sensitive, economical, and specific monitoring tools. Dugheri et al. [Contribution 8] developed an on-sample derivatization and GC-MS method for FA analysis in water using an SPME Arrow PDMS fiber, which is a large-bore SPME probe with greater phase volume and mechanical durability than standard SPME fibers. This approach offers a greener and more efficient “one-pot” analytical method by integrating commercially available cooling-assisted SPME with multiplexed SPME techniques for fully automated monitoring.

Chemical pest control using pesticides is widely practiced in agriculture and forestry, but concerns remain about the impact of environmental water pollution on human health and ecosystems. Zang et al. [Contribution 9] developed an Fe_3_O_4_/persimmon leaf magnetic biomass composite for pollutant removal and detection in water. They also establishes a method for analyzing four pesticides (trifluralin, triadimefon, permethrin, and fenvalerate) in environmental water samples using MSPE combined with GC-ECD. This novel biomass composite serves as an effective, sustainable adsorbent, promoting green analytical methods by repurposing waste biomass.

Sulfonamides are widely used in meat-producing livestock to prevent bacterial infections due to their effectiveness, stability, and affordability. However, their excessive or prolonged use may lead to residue accumulations in animal products, posing health risks to humans, including allergic reactions, drug resistance, teratogenicity, carcinogenicity, and mutagenicity. Liu et al. [Contribution 10] developed COPs mixed-matrix membranes by incorporating porous COPs into polyvinylidene fluoride polymer using Schiff base chemistry. They also established an ME/HPLC method for analyzing of six sulfonamides in human urine. This novel COP-based membrane effectively preconcentrates trace organic compounds from complex matrices.

Antineoplastic drugs cause genetic damage not only to cancer cells, but also to healthy cells, posing significant risks to both patients undergoing treatment and healthcare workers exposed occupationally. Dugheri et al. [Contribution 11] developed a rapid, automated, highly sensitive method to determine urinary concentrations of the DNA alkylating agents cyclophosphamide and ifosfamide using µSPE followed by UHPLC-MS/MS for biological monitoring. This µSPE-bases approach has become a powerful tool for occupational health and aligns with green analytical strategies.

Passive exposure to environmental tobacco smoke not only increases the risk of lung cancer and cardiovascular disease, but may also act as a stressor linked to neuropsychiatric and other conditions. To investigate this relationship, Kataoka et al. [Contribution 12] developed an online automated analysis system for measuring tobacco smoke exposure biomarkers, including nicotine and cotinine, alongside stress-related biomarkers, such as cortisol, serotonin, melatonin, dopamine, and oxytocin in saliva samples. The method employs IT-SPME with a Supel-Q PLOT capillary as the extraction device, followed by LC-MS/MS analysis. This non-invasive, simple, and highly sensitive technique has confirmed that tobacco smoke exposure acts as a stressor for non-smokers.

Finally, as editor of this Special Issue, I hope that the articles published will inspire new perspectives and ideas for further research.
